# Identification of a Novel Small RNA *srvg23535* in *Vibrio alginolyticus* ZJ-T and Its Characterization With Phenotype MicroArray Technology

**DOI:** 10.3389/fmicb.2018.02394

**Published:** 2018-10-05

**Authors:** Yiqin Deng, Youlu Su, Songlin Liu, Zhixun Guo, Changhong Cheng, Hongling Ma, Jinjun Wu, Juan Feng, Chang Chen

**Affiliations:** ^1^Key Laboratory of South China Sea Fishery Resources Exploitation and Utilization, Ministry of Agriculture and Rural Affairs, South China Sea Fisheries Research Institute, Chinese Academy of Fishery Sciences, Guangzhou, China; ^2^Key Laboratory of Tropical Marine Bio-resources and Ecology, Guangdong Provincial Key Laboratory of Applied Marine Biology, South China Sea Institute of Oceanology, Chinese Academy of Sciences, Guangzhou, China; ^3^Key Laboratory of Tropical Marine Bio-resources and Ecology, South China Sea Institute of Oceanology, Chinese Academy of Sciences, Guangzhou, China; ^4^Xisha/Nansha Ocean Observation and Research Station, South China Sea Institute of Oceanology, Chinese Academy of Sciences, Guangzhou, China

**Keywords:** small non-coding RNAs, *srvg23535*, *Vibrio alginolyticus*, identification, Phenotype MicroArray technology

## Abstract

Small non-coding RNAs (sRNAs) are important modulators of gene expression and are involved in the pathogenesis and survival of prokaryotes. However, few studies have been conducted with *Vibrio alginolyticus*, which limits our ability to probe the regulation of virulence and environmental adaptation by sRNAs in this opportunistic pathogen. In this study, the sRNA candidate *srvg23535* was identified in *V. alginolyticus* ZJ-T. The precise transcript end, secondary structure, and sequence conservation were determined. A *srvg23535* null mutant was constructed and characterized by using Phenotype MicroArray (PM) technology. *In silico* target prediction was conducted by IntaRNA and TargetRNA2. Subsequently, a 107 nt transcript was validated with a sigma70 promoter at the 5′ end and a Rho-independent terminator at the 3′ end. The sRNA *srvg23535* had four stem-loop structures and was conserved among *Vibrio harveyi, Vibrio parahaemolyticus*, and *Vibrio splendidus*. Deletion of *srvg23535* in *V. alginolyticus* ZJ-T led to a weaker utilization of D-mannose, D-melibiose, lactulose, and inosine as carbon sources but stronger utilization of L-cysteine as nitrogen source. Moreover, the *srvg2353* mutant showed stronger resistance to osmotic stress but weaker resistance to pH stress. Additionally, a total of 22 common targets were identified and several were related to the observed phenotype of the mutant. This study indicated that the novel sRNA, *srvg23535*, is conserved and restricted to *Vibrio* spp., affecting the utilization of several carbon and nitrogen sources and the response to osmotic and pH stress. These results extend our understanding of sRNA regulation in *V. alginolyticus* and provide a significant resource for the further study of the precise target mRNAs of *srvg23535*, which may provide targets for antibacterial therapeutic or attenuated vaccines against *Vibrio* spp.

## Introduction

*Vibrio alginolyticus*, a Gram-negative halophilic bacterium with worldwide distribution, is an opportunistic pathogen of marine animals and humans, which causes serious infections, such as otitis media, otitis externa, and gastroenteritis (Citil et al., 2015; Siddiqui et al., 2016). During invasion, pathogenic bacteria may be exposed to a variety of harsh environments, including high pH, high salinity, and starvation (Ulitzur, 1975; Roop et al., 2009). To adapt to rapid changes of environmental conditions, bacteria need to coordinate their gene expression quickly and precisely (Ruprecht et al., 2016). Many genes associated with infection and the ability of bacteria to survive in the host have been identified in *V. alginolyticus* (Ren et al., 2013; Xiong et al., 2014). However, empirical evidence of complex post-transcriptional regulation and coordinating gene expression that allow *V. alginolyticus* to adapt to the harsh environments is rare.

Small non-coding RNAs (sRNAs) are important post-transcriptional regulators in both eukaryotes and prokaryotes (Liu and Camilli, 2010). They usually function by base-pairing with mRNA to modulate stability and/or translation (Wagner and Romby, 2015). In bacteria, sRNAs have been reported to control a variety of biological processes, including metabolism, bacterial motility, biofilm formation, quorum sensing, iron homeostasis, and stress responses (Wagner and Romby, 2015; Liu W. et al., 2016), and, therefore, are associated with bacterial adaptation and virulence. Several reports have shown that *Vibrio* spp. encode sRNAs as well, which were mainly identified and studied in *Vibrio cholerae* and *Vibrio harveyi* (Nguyen and Jacq, 2014). These sRNAs include RyhB, which functions in iron homeostasis (Mey et al., 2005), MicX, which regulates the synthesis of outer membrane proteins (Davis and Waldor, 2007), and Qrrs, involved in quorum sensing (Tu and Bassler, 2007). Nevertheless, the function of sRNA in *V. alginolyticus* is poorly understood (Luo et al., 2016). The potential regulatory function of sRNAs in *V. alginolyticus* could be important in environmental adaptation, which probably affects the virulence of bacteria (Iustman et al., 2015).

The prediction and validation of bacterial sRNAs are two necessary steps before studies of function. With the development of high-throughput sequencing and bioinformatics, genome-wide searches based on the biocomputational prediction of non-coding RNA genes are widely used in the prediction of sRNAs both in eukaryotes and prokaryotes, and even archaea (Livny and Waldor, 2007). Predicted sRNAs are subsequently subjected to experimental physical validation, including Northern blot analysis, qPCR, and 5′ and 3′ rapid amplification of cDNA ends (RACE) (Sharma and Vogel, 2009). Given that most functional sRNAs are expressed independently, relying on a stable secondary structure, and are usually conserved among different species or even different genus, the study of promoter and terminator identification and conservation analysis are often conducted to promote function research (Argaman et al., 2001; Chen et al., 2002).

The Phenotype MicroArray (PM) platform for global analysis of cellular phenotypes was first developed by Bochner et al. (2001). The technology has been used to monitor most aspects of the cell function, including the utilization of nutrients, transportation, and catabolization (PM1-8), and sensitivity to environmental stresses, such as osmolytes, pH, and chemical agents (PM9-20) (Bochner, 2009). It has been developed to directly assay the effects of genetic changes in cells (Tohsato and Mori, 2008). For example, (p)ppGpp and c-di-GMP were found to be involved in antibiotic resistance in *Mycobacterium smegmatis* by analyzing the wild type and strains lacking the gene for either (p)ppGpp synthesis or c-di-GMP synthesis by PM analysis (Miller, 1998).

During our preliminary research, 194 sRNA candidates were predicted through RNA-seq by the presence or absence of the RNA molecular chaperone Hfq in *V. alginolyticus* ZJ-T (Yiqin et al., 2016). Consequently, several of them were identified by Northern blotting or qPCR. In this study, we reported the identification of *srvg23535*, which is novel and conserved among *Vibrio* spp. Additionally, we characterized its phenotype in the utilization of different carbon and nitrogen sources, and its response to osmotic and pH stress by using PM technology, and then predicted its genetic targets *in silico*. These data will enrich the study of sRNAs in *V. alginolyticus*, and help to discover more about the environmental adaptation of *V. alginolyticus*, which may provide new insights into its pathogenesis and assist in providing new antibacterial therapeutic targets or attenuated vaccines for *Vibrio* spp.

## Materials and Methods

### Bacterial Strains, Plasmids, and Growth Conditions

The bacterial strains and plasmids used in this study are listed in **Table 1**.

**Table 1:** Strains and plasmids used in this study.

Strains or plasmids	Relevant characteristics	Sources
***V. alginolyticus***		
ZJ-T	Ap^r^ (ampicillin resistant), translucent/smooth variant of wild strain ZJ-51 (Xiaochun et al., 2017); isolated from diseased *Epinephelus coioides* off the Southern China coast	Chang et al., 2009
ZJ-T-Δ*srvg23535*	Apr; ZJ-T carrying an deletion of *srvg23535*	This study
***E. coli***		
П3813	Emr^r^, Tc^r^, *lacIQ, thi1, supE44, endA1, recA1, hsdR17, gyrA462, zei298::tn10[Tc], ΔthyA::(erm-pir116)*; the intermediate host of suicide vector pSW7848	Le Roux et al., 2007
GEB883	Ery^r^, Tet^r^, WT *E. coli* K12 *ΔdapA::erm pir RP4-2 ΔrecA gyrA462, zei298::Tn10*; donor strain for conjugation	Nguyen et al., 2018
**Plasmids**		
pSW7848	Cmr; suicide vector with an R6K origin, requiring the Pir protein for its replication, and the *ccdB* toxin gene	Val et al., 2012
pSW7848-Δ*srvg23535*	Cmr; pSW848 containing the mutant allele of Δ*srvg23535*	This study

*V. alginolyticus* was cultured in LBS [Luria–Bertani (LB) with 2% additional NaCl] medium at 30°C. *Escherichia coli* strains were cultured in LB medium (BD, United States) supplemented with appropriate antibiotics at 37°C. For the selection of transconjugants, TCBS medium (BD, United States) was used with 0.2% D-glucose and 5 μg ml^-1^ chloramphenicol (Cm). To select transconjugants that have undergone plasmid excision and allelic exchange, expression of the *ccdB* gene carried by the pSW7848 plasmid was induced by adding 0.2% L-arabinose to the medium. This gene encodes a topoisomerase which is lethal to Gram-negative bacteria (Loris et al., 1999).

### Northern Blot Assay

The sRNA, *srvg23535*, was named according to the upstream gene locus. It was predicted by RNA-seq of *V. alginolyticus* ZJ-T and the *hfq* mutant strain Δ*hfq*-T with the following four features: (i) the sRNA was expressed from an intergenic regions and the size was between 50 and 500 nt; (ii) the expression level was at least half of but not the same as that of the flanking genes; (iii) there was a sigma70 promoter at the 5′ end and a Rho-independent terminator at the 3′ end; and (iv) the secondary structure was stem loop structure. Subsequently, Northern blotting was conducted as described by Toffano-Nioche et al. (2012) with some modification to confirm the transcription of *srvg23535*. Briefly, *V. alginolyticus* ZJ-T was cultured in LBS and Zobell medium at the same time and bacterial cells were collected for RNA extraction from different time points along the growth curves. The RNA was separated with 8% PAGE and transferred to a Hybond N^+^ membrane. An oligonucleotide probe, NB-*srvg23535* (**Supplementary Table S1**), was labeled at the 3′ end using terminal transferase (Fermentas, United States) and [α-32p] dCTP. RiboRuler Low Range RNA Ladder (Fermentas, United States) was run alongside the samples to allow an estimation of the transcript size. Briefly, within a certain size range, the nucleic acid migration distance (electrophoretic mobility) is inversely proportional to the logarithm of its size (Bishop et al., 1967; Gough and Gough, 1984). Thus, according to the size of different marker’s bands and their migration distance, a curve can be drawn with the migration distance as the horizontal axis and the logarithm of the size as the vertical axis. Then, according to the migration distance of the target sRNA, the size of it can be estimated. 5.0S RNA was used as a loading control with the NB-5.0S RNA probe (**Supplementary Table S1**).

### RACE

The corresponding 5′ and 3′ end of *srvg23535* were identified by 5′ and 3′ RACE, respectively. Briefly, *V. alginolyticus* ZJ-T was cultured overnight in LBS medium at 30°C with vigorous shaking. The overnight culture was diluted to OD_600_ 0.01 with fresh LBS medium and grown until OD_600_ 0.8. Total RNA was isolated by using RNAprep Pure Cell/Bacteria Kit (TIANGEN, China). 5′ and 3′ RACE was conducted by using a SMARTer^®^ RACE 5′/3′ Kit (Clontech, United States) (**Supplementary Table S1**). Finally, at least 12 clones of 5′ or 3′ RACE action were sequenced to determine the ends of *srvg23535*.

### Promoter, Terminator Characterization, and Novelty Assessment

According to the transcript sequence, the sigma70 promoter was predicated by BPROM (Solovyev and Salamov, 2011) searching the region 200 nt upstream of the 5′ end, while the Rho-independent terminator was predicated by ARNold (Lesnik et al., 2001) searching the region 200 nt downstream of the 3′ end. The transcript sequence was blasted into the Rfam database to assess the novelty (Griffithsjones et al., 2003).

### Secondary Structure Characterization and Phylogenetic Analysis

The MFOLD (Zuker et al., 1999) program was used to predicate the secondary structure of *srvg23535* based on the lowest folding energy. The sequence including the transcript and the terminator were blasted against the representative genomes of microbes. Then multiple sequences alignment was conducted using ClustalW. The phylogenetic tree was constructed from the aligned sequences using Kimura 2-parameter model with the neighbor-joining method, bootstrapped 1,000 times via MEGA6.0 software.

### *In silico* Analysis of Target Genes

The target genes of *srvg23535* were predicted by using Web-based programs: IntaRNA (Siqueira et al., 2016) and TargetRNA2 (Kery et al., 2014). The hybridization between the sRNA transcript sequence and the sequence comprising 150 nt upstream until 150 nt downstream of the start codon of each annotated gene was screened in the genome of *V. alginolyticus* ZJ-T. To consider an interaction as positive, we used the corresponding *p* < 0.05 of both programs, and energy < -13 kcal/mol with IntaRNA and synonym energy < -8 kcal/mol with TargetRNA2 were taken as the threshold. Finally, the common predictions of both programs were confirmed as the target candidates.

### Gene Disruption

**FIGURE 1:**
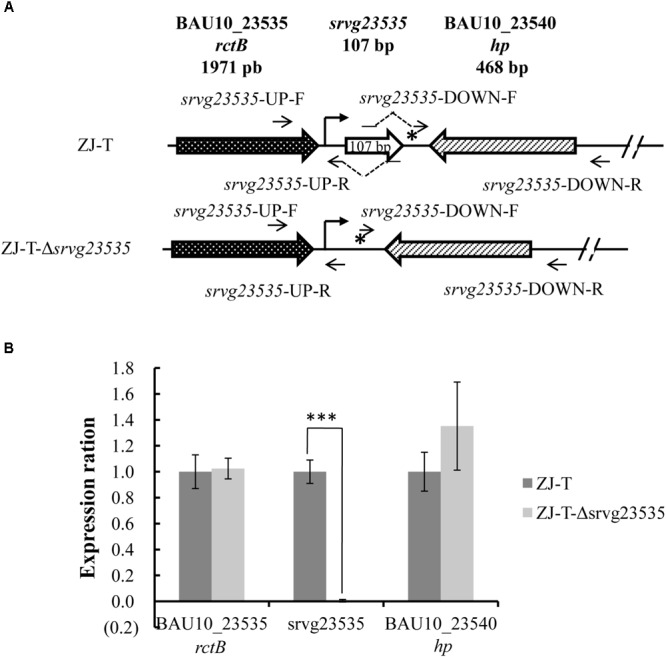
Structure of the *srvg23535* locus and neighboring gene expression in *V. alginolyticus* ZJ-T and the mutant strain. **(A)** The sRNA *srvg23535* is downstream of the *rctB* gene (locus ID = BAU10_23535) and upstream of a hypothetical protein gene (locus ID = BAU10_23540). A 107 nt transcript was determined by RACE. A putative Sigma70 promoter identified upstream of *srvg23535* is indicated by an arrow and a Rho-independent terminator identified downstream of *srvg23535* is indicated by a star. A total of 155 nt, from the 46 bp before the 5’ end to the 2 bp after the 3’ end was deleted, giving rise to ZJ-T-Δ*srvg23535*. **(B)** Relative expression of *srvg23535*, the upstream gene (*rctB*) and the downstream gene (*hp*) in the mutant strain compared to the wild type. Relative expression (normalized to the wild-type level for each gene) was determined by qPCR. Error bars correspond to the standard errors from three biological replicates. Statistically significant differences are indicated (^∗∗∗^*p* < 0.001).

To generate the sRNA disruptant, the sequence from 46 bp before the 5′ end to 2 bp after the 3′ end was deleted from the chromosome of *V. alginolyticus* ZJ-T. The deletion was constructed by homologous recombination as described before with some modification (Yiqin et al., 2016). Briefly, two flanking fragments of *srvg23535* (**Figure 1A**) were amplified with two pairs of primers, *srvg23535*-UP-F and -R and *srvg23535*-DOWN-F and -R, respectively, and the linearized pSW7848 was amplified with pSW7848-F and -R (**Supplementary Table S1**). *srvg23535*-UP-F and *srvg23535*-DOWN-R contained overlapping extensions with pSW7848-R and -F, respectively, and *srvg23535*-UP-R contained overlapping extensions with *srvg23535*-DOWN-F. The two flanking fragments were further assembled into the linearized pSW7848 by using a ClonExpress Multis One Step Cloning Kit (Vozyme, China), generating the recombinant plasmid pSW7848-Δ*srvg23535* comprising the 1,084 bp upstream and 1,105 bp downstream regions of *srvg23535* (**Table 1**), using *E. coli* П3813 as an intermediate host. The recombinant plasmid was transferred by conjugation from strain GEB883 (**Table 1**) to *V. alginolyticus* ZJ-T before allelic exchange as described above. The sRNA disruptant was then confirmed by sequencing and the strain was named ZJ-T-Δ*srvg23535* (**Figure 1** and **Table 1**).

### Quantitative RT-PCR (qPCR)

qPCR was conducted to test the potential effect of the *srvg23535* deletion on neighboring gene expression (potential polar effects) as described below (**Supplementary Table S1**). Three single colonies of strains ZJ-T and ZJ-T-Δ*srvg23535* were inoculated overnight in three independent cultures with LBS medium. Overnight cultures were then diluted to OD_600_ 0.01 in LBS fresh medium and bacterial cells were collected by centrifugation when grown to early exponential phase (OD_600_ 0.5). All reagents were from Takara Bio Inc. (Japan). Total RNA was isolated by using RNAiso Plus, and 1 μg of RNA was DNase I-treated and reverse transcribed with PrimeScript^TM^ RT reagent Kit with gDNA Eraser. PCR amplification was then carried out on a LightCycler480 (Roche, Switzerland) by using SYBR Premix Ex Taq^TM^ II with primers specific to the genes to be assayed. Three housekeeping genes (*recA, uvrA*, and *gyrA*) were used as internal controls (**Supplementary Table S1**). Three technological assessments were conducted on each biological replicate. Relative gene expression was calculated by the 2^-ΔΔCt^ method (Livak and Schmittgen, 2001) and normalized to the wild-type ZJ-T value. Statistical significance was determined by the Student’s *t*-test (^∗^*p* < 0.05, ^∗∗^*p* < 0.01, ^∗∗∗^*p* < 0.001).

### PM Assay

Phenotype MicroArray technology was used to identify the function of *srvg23535* in the utilization of different carbon sources, nitrogen sources, response to osmotic stress, and response to pH stress with the plates PM01, PM03, PM09, and PM10, respectively (Bochner et al., 2001). The test components are shown in **Supplementary Table S2**. All the substrates tested were pre-dispensed and dried in 96-well plates, requiring only inoculation with bacteria suspended in a buffer containing a dye, such as tetrazolium violet. During growth, bacterial metabolism leads to the irreversible reduction of the dye with the production of a purple color that can be read as the change in absorbance over time (Khatri et al., 2013). The measurements of growth were conducted at 590 nm (Blumenstein et al., 2015).

The wild type and the *srvg23535* disruption cells were streaked on LBS agar plates and grown overnight at 30°C. All PM procedures were performed according to the protocol “PM Procedures for *E. coli* and other GN Bacteria” (Biolog, Inc., 26 August 2005; **Supplementary File S1**) with some modification because *V. alginolyticus* achieved better growth with 3% NaCl. For each strain and each biological replicate, 260 μl Dye Mix A and 4,075 μl water containing 14% NaCl were added to the inoculating fluid of 1.2 × IF-0, resulting in a final volume of 26 ml 1.0 × IF-0. In addition, 240 μl Dye Mix A and 3,760 μl water containing 14% NaCl were added to the inoculating fluid of 1.2 × IF-10 and resulting in a final volume of 24 ml 1.0 × IF-10. Cells were transferred and suspended into 26 ml of 1.0 × IF-0 to achieve 85% T (transmittance) in the BIOLOG Turbidimeter. Each well of the PM01 plate (carbon sources) was inoculated with 100 μl of the 85% T cell suspension. As the PM03 experiment required an appropriate carbon source, a stock solution of 2 M sodium succinate was used as an additive as recommended in the PM procedures for Gram-negative bacteria. Finally, 120 μl of the 2 M sodium succinate (100×) was added to 12 ml of the 85% T cell suspension prepared for PM01 and mixed completely but gently. Then, each well of the PM03 plate (nitrogen sources) was inoculated with 100 μl of the prepared cell suspension. For PM09 and PM10 experiments, 120 μl of the 85% T cell suspension prepared for PM01 was transferred into the prepared 24 ml 1.0 × IF-10 + dye (1:200 dilution) and mixed completely but gently. Then, each well of the PM09 and PM10 plates (the response to osmotic and pH stress, respectively) were inoculated with 100 μl of the prepared cell suspension. All PM plates were sealed with parafilm and inoculated in the incubator at 30°C. OD_590_ was measured at regular time intervals using a Multiskan Ascent plate reader (Thermo Fisher Scientific, United States) to indicate the conversion of the tetrazolium dye for 5 days. Finally, three biological replicates were conducted and the read-outs were analyzed with graphs. A one-way analysis of covariance (ANCOVA) was used to analyze the effect of treatment (*srvg23535* deletion) on the OD590 with time as a covariate.

## Results

### Computational Prediction and Experimental Identification of *srvg23535* in *V. alginolyticus*

**FIGURE 2:**
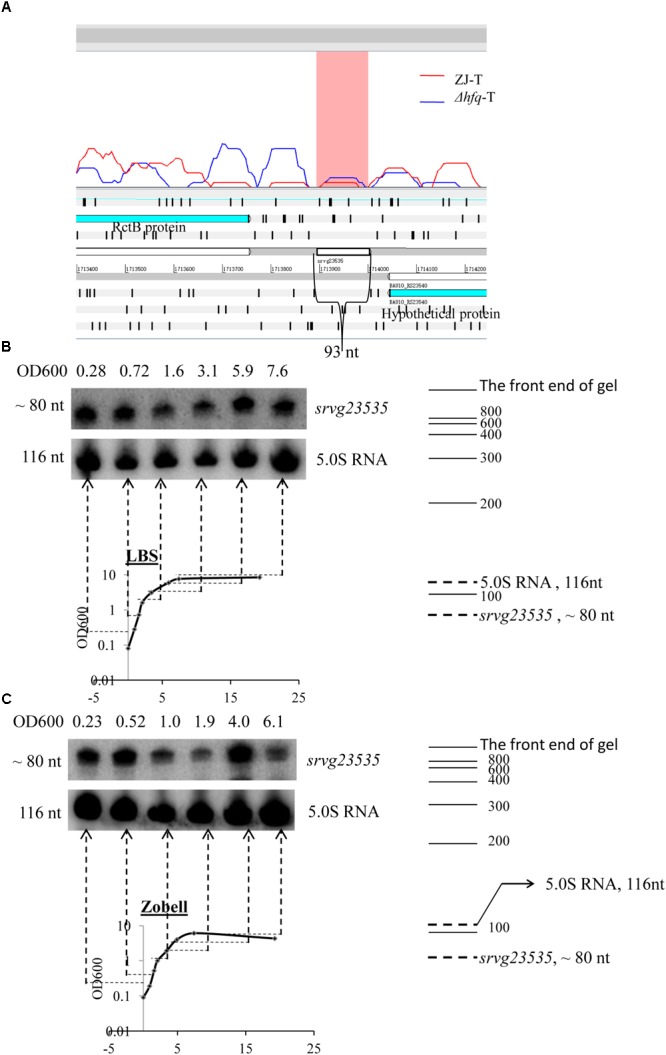
Locus feature and the expression of *srvg23535*. **(A)** An Artemis window shows the expression of *srvg23535* flanking by *rctB* and an *hp* gene. The data was adapted from the RNA-seq results (Yiqin et al., 2016). **(B)** Northern blot of *srvg23535* in LBS medium. The culture OD_600_ for each sample is indicated above each lane and the growth curve is presented underneath. The name of the sRNA is indicated on the right and the approximate size of the bands (determined by comparison with the position of RNA markers of known size) is indicated on the left. 5.0S RNA was used as a loading control. **(C)** Northern blot of *srvg23535* in Zobell medium. The legend is the same as **(B)**.

The sRNA candidate *srvg23535* is shown in an Artemis window (**Figure 2A**). It is flanked by a forward expressed translation elongation factor gene *rctB* (BAU10_23535) and a reverse expressed hypothetical protein gene (BAU10_23540) with a size of 93 nt. Only a single band was detected and its apparent size of around 80 nt in the Northern blot is smaller than the size of 93 nt deduced from the RNA-seq experiment (**Figures 2B,C**). As the bacteria grew, *srvg23535* was expressed consistently both in LBS and Zobell medium (**Figures 2B,C**).

### Transcriptional Start and End Points of *srvg23535*

**FIGURE 3:**
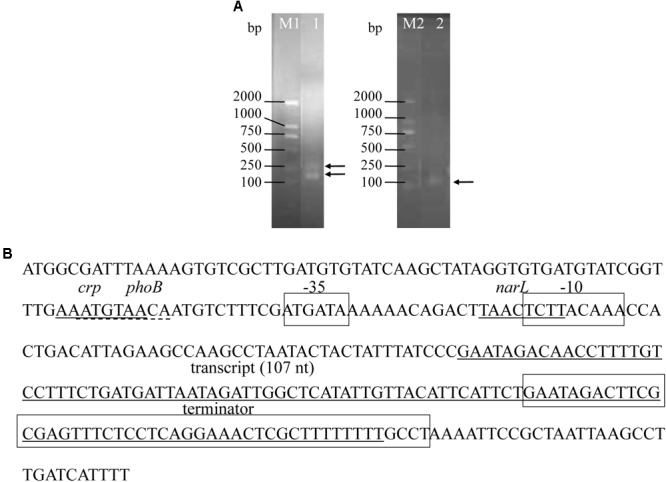
Precise transcriptional start and end points of *srvg23535* were determined by the RACE experiments, and the promoter and terminator were predicated. **(A)** PCR results of 5′- and 3′-RACE. The bands were indicated by arrows. Lane M1: DNA Marker DL2000, it was cropped from **Supplementary Figure S1A** lane M; Lane 1: 5′-RACE of *srvg23535*, it was cropped from **Supplementary Figure S1A** lane 3; Lane M2: DNA Marker DL2000, it was cropped from **Supplementary Figure S1B** lane M; Lane 2: 3′-RACE of *srvg23535*, it was cropped from **Supplementary Figure S1B** lane 6. **(B)** Transcript size of *srvg23535* was 107 nt. The first solid line covers the sequence of “AAATGTAA,” and indicates the *crp* transcriptional regulate factor; the first dashed line covers the sequence of “ATGTAACA,” and indicates the *phoB* transcriptional regulate factor; the second solid line covers the sequence of “TAACTCTT,” and indicates the *narL* transcriptional regulate factor; the third solid line covers the sequence form “GAATAGACAA” to “GCTTTTTTTT,” and indicates the total transcript of *srvg23535*; the first frame covers the sequence of “ATGATA,” and indicates the –35 box; the second frame covers the sequence of “TCTTACAAA,” and indicates the –10 box; the third frame covers the sequence from “GAATAGACTT” to “TTTTTTGCCT,” and indicates terminator of *srvg2353*5.

Two bands were detected for 5′ RACE and one band was found for 3′ RACE (**Figure 3A** and **Supplementary Figure S1**). However, the sequence results showed that the larger band of 5′ RACE was by-product and non-specific product, and only one transcript was defined by RACE (**Figure 3B**), which was consistent with the results obtained from Northern blotting. The most abundant *srvg23535* was 107 nt in length (**Figure 3B**), which is bigger than that deduced from RNA-seq (93 nt) and estimated by Northern blotting (around 80 nt).

### Promoter, Terminator, and Novelty Analysis

One sigma70 promoter was predicated by BPROM and one Rho-independent terminator was predicated by ARNold (**Figure 3B**). Apart from the -10 and -35 boxes, three (a *crp*, a *phoB*, and a *narL*) transcriptional regulatory factors were predicted in the promoter (**Figure 3B**). Novelty assessment by blasting into the Rfam database showed that the sRNA was found first.

### The Secondary Structure and the Conservation of *srvg23535*

**FIGURE 4:**
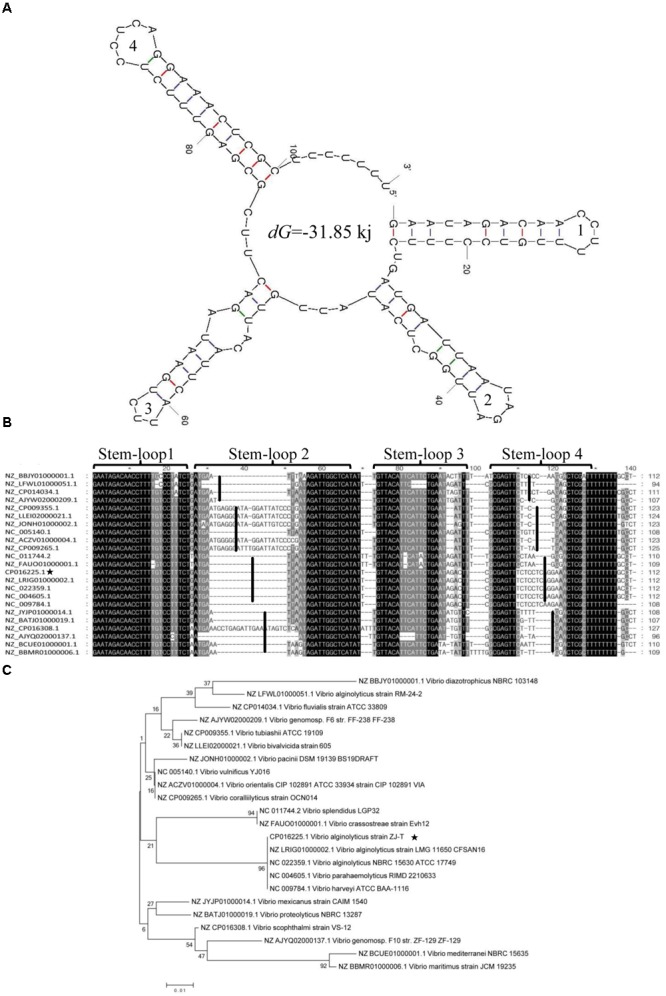
Secondary structure, multi-sequence alignment and phylogenetic analysis of *srvg23535*. **(A)** The *srvg23535* was consisted of four stem-loop structures with a *dG* of –31.85 kJ, and the stem-loops were numbered. **(B)** The multi-sequence alignment result. The sequences were divided into four clusters by stem-loop 2 and 4. The sequences of stem-loop 1 and 3 were highly similar to other homologs. **(C)** The phylogenetic tree of *srvg23535* with representative microbes aligned by using ClustalW. The numbers on the branch points mean the relative bootstrap (%, Bootstrap values ÷ 1000 × 100). The bar indicates 0.01 substitutions per sequence position.

A total of four stem-loop structures with a *dG* of -31.85 kJ may facilitate the high stability of *srvg23535* (**Figure 4A**). Stem-loop 4, which probably acts as a putative transcriptional terminator, was predicted near the 3′ end region of *srvg23535*. The multi-sequence alignment of *srvg23535* against representative genomes of microbes indicated that the sequences of stem-loop 1 and 3 had high similarity to other homologs. However, the transcript sequences were divided into four clusters by the sequence of stem-loop 2 and 4, which were consistent with the phylogenetic tree (**Figure 4B**). Furthermore, the phylogenetic tree showed that the *srvg23535* was conserved among *V. harveyi, Vibrio parahaemolyticus*, and *Vibrio splendidus*, but not present in *Vibrio tapetis, Vibrio aesturianus, V. cholerae* or in species outside the *Vibrio* (**Figure 4C**).

### sRNA Target Prediction

A total of 22 common targets were identified under the parameters set (**Table 2** and **Supplementary Table S3**). Then we parsed the predicted mRNA targets based on their GO annotation and KEGG pathway. The results showed that the 22 targets were involved in a series of cellular processes, including metabolism, virulence, transport, and bacterial chemotaxis (**Supplementary Table S3**). However, six of the target genes were categorized as “hypothetical proteins” (**Table 2** and **Supplementary Table S3**).

**Table 2:** Common tartets predication by IntaRNA and TargetRNA2.

Target gene name	Gene	Products	KEGG
BAU10_07960	*–*	Type III secretion regulator ExsE	–
BAU10_08945	*–*	Threonylcarbamoyl-AMP synthase	K07566//tRNA threonylcarbamoyladenosine biosynthesis protein
BAU10_02460	*dxs*	1-Deoxy-D-xylulose-5-phosphate synthase	K01662//1-deoxy-D-xylulose-5-phosphate synthase [EC:2.2.1.7]
BAU10_15400	*–*	ABC transporter permease	K02034//peptide/nickel transport system permease protein
BAU10_07225	*copB*	ATPase P	K01533//Cu2+-exporting ATPase
BAU10_22390	*araC*	Aminotransferase	K11325//L-cysteine/cystine lyase; K04127//isopenicillin-N epimerase [EC:5.1.1.17]
BAU10_14655	*fre, ubiB*	NAD(P)H-flavin reductase	K05368//aquacobalamin reductase/NAD(P)H-flavin reductase [EC:1.16.1.3 1.5.1.41]
BAU10_17580	*aas*	Acyl-phosphate glycerol 3-phosphate acyltransferase	K00680//[EC:2.3.1.-]; K05939//acyl-[acyl-carrier-protein]-phospholipid *O*-acyltransferase/long-chain-fatty-acid–[acyl-carrier-protein] ligase [EC:2.3.1.40 6.2.1.20]
BAU10_22080	*mcp*	Chemotaxis protein	K03406//methyl-accepting chemotaxis protein
BAU10_10410	*gcvR*	Glycine cleavage system transcriptional repressor	K03567//glycine cleavage system transcriptional repressor
BAU10_10195	*cheZ*	Protein phosphatase	K03414//chemotaxis protein CheZ
BAU10_18400	*maa*	Maltose acetyltransferase	K00661//maltose *O*-acetyltransferase [EC:2.3.1.79]
BAU10_22015	*kdpD*	Histidine kinase	K07646//two-component system, OmpR family, sensor histidine kinase KdpD [EC:2.7.13.3]
BAU10_16085	*opsX*	Glycosyl transferase	K12982//heptosyltransferase I [EC:2.4.-.-]
BAU10_19325	*–*	Flavodoxin	–
BAU10_03555	*–*	FAD-dependent oxidoreductase	K00540//[EC:1.-.-.-]
BAU10_03760	*–*	Hypothetical protein	*–*
BAU10_19400	*–*	Hypothetical protein	*–*
BAU10_18585	*–*	Hypothetical protein	*–*
BAU10_07805	*–*	Hypothetical protein	*–*
BAU10_19060	*–*	Hypothetical protein	*–*
BAU10_02660	*–*	Hypothetical protein	*–*

### Construction of srvg23535 Null Mutant

A gene disruptant of *srvg23535*, from 46 bp before the 5′ end to 2 bp after the 3′ end, was constructed (**Figure 1A**). It was important to verify whether the deletion had a polar effect on the neighboring genes, thus we checked the expression by qPCR of all three genes (upstream, *srvg23535*, and the downstream gene) in wild type and ZJ-T-Δ*srvg23535*. As shown in **Figure 1B**, a significant difference of expression was observed only in the case of *srvg23535*: expectedly expression could not be detected in the mutant.

### Functional Analysis of *srvg23535*

**FIGURE 5:**
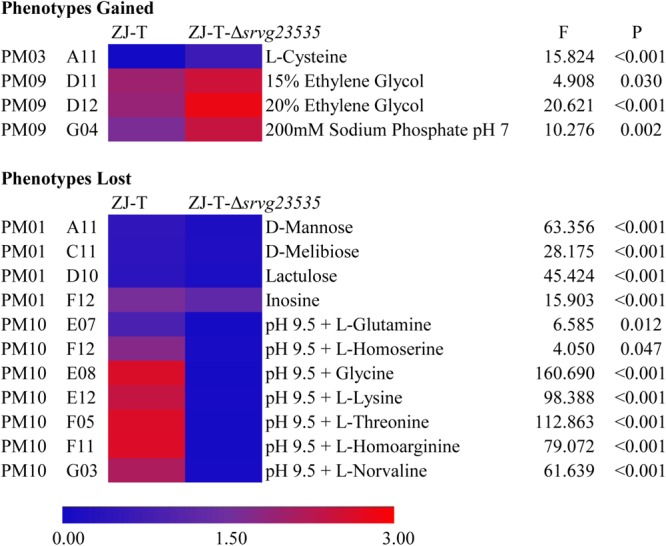
Gained and lost phenotypes in the absence of *srvg23535* are described with a heat map (*F*- and *P*-values correspond to the results of one-way ANCOVA).

In total, 95 different carbon sources (PM01), 95 nitrogen sources (PM03), and tolerances to different osmolytes (PM09) and pH (PM10) were detected to identify the comprehensive role of *srvg23535* by using PM technology. General results are shown in **Supplementary Figures S2–S5**, respectively. Significantly different phenotypes between the wild type and the mutant strain are described in the form of a heat map shown in **Figure 5**. The heat map was created with OD590 of the maximum of different time points. The details of phenotype differences are shown in **Supplementary Table S4** and their original data is shown in **Supplementary File S2**.

#### Carbon Utilization

Both the wild type ZJ-T and the *srvg23535* mutant strain ZJ-T-Δ*srvg23535* were able to utilize 60.0% (57/95) of the tested carbon sources. Compared to the wild type, the mutant has a longer lag phase or lost viability during stationary phase when bacteria were cultured with D-mannose, D-melibiose, lactulose or inosine (PM01 wells A11, C11, D10, and F12, respectively; one-way ANCOVA, *F* = 63.356, 28.175, 45.424, 15.903, respectively, and all *P* < 0.001) as carbon sources (**Figure 5, Supplementary Figure S2**, and **Supplementary Table S4**).

#### Nitrogen Utilization

Generally, ZJ-T and ZJ-T-Δ*srvg23535* almost showed the same availability and utilization of the tested nitrogen sources in PM03, except that ZJ-T-Δ*srvg23535* gained sharp growth after 3 days with L-cysteine as a nitrogen source (PM03 well A11; one-way ANCOVA, *F* = 15.824 and *P* < 0.001), whereas ZJ-T could not grow (**Figure 5, Supplementary Figure S3**, and **Supplementary Table S4**).

#### Osmolytes Tolerance

The PM09 data showed that as the concentration of ethylene glycol increased (PM09 wells D09 to D12), ZJ-T-Δ*srvg23535* obtained a higher viability at stationary phase, showing a stronger tolerance to ethylene glycol, especially to 15% and 20% ethylene glycol (PM09 well D11 and D12; one-way ANCOVA, *F* = 4.908 and 20.621, respectively, and *P* = 0.030 and <0.001, respectively) (**Figure 5** and **Supplementary Table S4**). The mutant obtained a higher viability at stationary phase when exposed to 200 mM sodium phosphate at a pH = 7 (PM09 well G04; one-way ANCOVA, *F* = 10.276, and *P* = 0.002) (**Figure 5** and **Supplementary Table S4**).

#### Survival in Different pH Environments

PM10 data indicated that, compared to the wild type, the mutant had a longer lag phase when grown at pH 9.5 with L-glutamine or L-homoserine (PM10 wells E07 and F12, respectively; one-way ANCOVA, *F* = 6.585 and 4.050, respectively, and *P* = 0.012 and 0.047, respectively) (**Figure 5** and **Supplementary Table S4**), and totally lost growth at pH 9.5 with glycine, L-lysine, L-threonine, L-homoarginine or L-norvaline (PM10 wells E08, E12, F05, F11, G03, respectively; one-way ANCOVA, *F* = 160.690, 98.388, 112.863, 79.072, and 61.639, respectively, and all *P* < 0.001) (**Supplementary Figure S5** and **Supplementary Table S4**).

## Discussion

We used an RNA-seq strategy to identify candidate sRNAs of *V. aglinolyticus* ZJ-T and successfully identified *srvg23535*. Northern blotting showed that *srvg23535* was around 80 nt and displayed growth-phase-independent expression both in LBS and Zobell medium, indicating that *srvg23535* would not be functional phase-dependent probably without involving quorum sensing and biofilm formation (Nguyen and Jacq, 2014). Subsequently, the precise boundary of *srvg23535* was determined by using 5′ and 3′ RACE which identified a 107 nt transcript. The smaller transcript size estimated from Northern blot is probably the mature form of *srvg23535* since RNA processing plays a critical role in gene regulation (Duan et al., 2007). Or, it may probably be caused by the size calculation method of Northern blot which has limitation in measure of low or high molecular weight of fragments (Bishop et al., 1967; Gough and Gough, 1984). The Rfam database is a collection of RNA families (Kalvari et al., 2017), which can be used to assess the novelty of sRNAs. By this approach, *srvg23535* was showed to be first found, leading it more valuable to be characterized.

It has become clear that sRNA can have a conserved sequence and structure among homologs, resulting in a comprehensive identification of sRNAs by using comparative genomics (Peer and Margalit, 2011). According to the transcript determined by RACE, the sRNA conservation was analyzed with a phylogenetic tree. The result showed that *srvg23535* is conserved among several *Vibrio* species, such as *V. harveyi, V. parahaemolyticus*, and *V. splendidus*, but not found in *V. tapetis, V. aesturianus*, and *V. cholerae* or outside the *Vibrio* spp. Similarly, Tsai et al. (2014) have identified seven novel sRNAs in *Deinococcus radiodurans* and conservation analysis confirmed that the sRNAs were restricted to the Deinococcaceae family. However, although sRNAs have a similar sequence, they may perform different roles in different species with low similarity in the target-binding regions (Peer and Margalit, 2011). Thereby, *srvg23535* is identified among several *Vibrio* spp., the sequence and the stem-loop structures are divided into four clusters probably relating to different functions in different species.

Based on the transcript sequence, the promoter was characterized. Three transcriptional regulatory factors (a *crp*, a *phoB*, and a *narL*) were predicted in the sigma70 promoter, indicating that *srvg23535* could be activated by cAMP, phosphorylation, and nitrite and nitrate ions. It has been shown that the cAMP receptor protein (Crp) binds to cAMP, which causes a conformational change that allows Crp to bind tightly to the *crp* site in the promoter of the genes it controls. These genes are primarily involved in the transport and catabolism of sugars and amino acids (De and Gottesman, 2009). De and Gottesman (2009) showed that CyaR sRNA repressed the expression of *ompX, yqaE, nadE*, and *luxS* via Crp activation, providing a link between catabolite repression, quorum sensing, and nitrogen assimilation in *E. coli*. PhoB is a response regulator of the two-component signal transduction system. When PhoB is activated by phosphorylation, it binds to a *pho* box, such as *phoB*, in the promoter thereby promoting the transcription of genes whose products are involved in phosphorus uptake and metabolism (Neidhardt, 1996). NarL is triggered by nitrite and nitrate ions. Phosphorylated NarL binds to specific sequences within target promoters to regulate transcription initiation (Browning et al., 2004). These results suggest that *srvg23535* is probably involved in the transport and metabolism of carbon, nitrogen, and phosphorus sources.

Phenotypic regulation of *srvg23535* was systematically analyzed using PM technology between the wild type and the deletion mutant strain. In addition, target prediction was conducted to link phenotypic regulation and genetic regulation. sRNAs have been extensively reported to be involved in a variety of cellular processes (Gripenland et al., 2010; Papenfort and Vogel, 2014; Wagner and Romby, 2015). In this study, we found that the *srvg23535* mutant displayed growth defects when bacteria were cultured with D-mannose, D-melibiose, lactulose or inosine as the carbon sources. The absence of *srvg23535* may affect the transport or/and the degradation of these carbon sources. However, only a maltose acetyltransferase coding gene *maa* (BAU10_18400) was predicated as the common target involved in carbon metabolism. The mutant gained growth sharply after 3 days when the bacteria were cultured with L-cysteine as a nitrogen source, whereas the wild type could not grow in this condition. Consistent with such a phenotype, the target gene was predicted to be *araC* (BAU10_22390), which encodes an aminotransferase and is involved in L-cysteine lyase. In addition, the regulation of the utilization of carbon and nitrogen sources may also relate to the promoter feature as described before. During invasion and survival in the host, bacteria are exposed to different conditions (Ulitzur, 1975). A total of 44 sRNAs in *Geobacillus thermoleovorans* were found to be involved in high-temperature adaptation by Tan and Alam (2010). Here, we found that the *srvg23535* mutant showed a stronger tolerance to 15% ethylene glycol, 20% ethylene glycol, and 200 mM sodium phosphate at pH 7. However, compared to the wild type, the mutant had a weaker survival or even totally lost growth at pH 9.5 with L-glutamine, L-homoserine, glycine, L-lysine, L-threonine, L-homoarginine or L-norvaline. The two-component system and ABC transporter system are widely reported to regulate osmotic stress (Mizuno and Mizushima, 1990; Yan et al., 2013). Additionally, the two-component system is also reported to be involved in pH stress (Nakayama and Watanabe, 1995). Three genes, including an ABC transporter permease encoding gene (BAU10_15400, ABC transporter system), a chemotaxis protein-encoding gene (BAU10_22080, two-component system), and a histidine kinase encoding gene (BAU10_22015, two-component system) were predicted as being regulated by *srvg23535* and probably contributed to a stronger resistance to ethylene glycol osmotic stress and phosphate toxicity, and a weaker survival in different pH environments. Furthermore, *exsE*, a type III secretion regulator, was predicated as the target of *srvg23535* which indicates that *srvg23535* is probably involved in the pathogenicity of *V. alginolyticus*. ExsE has been reported as a regulator of the expression of type III secretion system, which generally contributes to the adhesion in pathogens (Rietsch et al., 2005; Erwin et al., 2012; Liu J. et al., 2016). In *Vibrio* species, including *V. alginolyticus, exsE* has been suggested to be a negative regulator of T3SS expression (Erwin et al., 2012; Liu J. et al., 2016).

Pathogens must adapt to various conditions to invade and survive in the host with a series of strategies by precise gene expression (Wagner and Romby, 2015). The regulation of metabolism and stress response are reported to be two main strategies of bacterial environmental adaptation (Yiqin et al., 2016). For example, *prfA* was reported to regulate glucose-1-phosphate utilization which affected the virulence of *Listeria monocytogenes* (Makarova et al., 2011). *Salmonella* spp. have evolved a variety of acid resistance mechanisms, including acid tolerance response (Van, 2010), acid resistance (Handa et al., 2000), and changes in cell membrane composition (Scherzinger et al., 1991) to survive in acid conditions, which affect their virulence. Therefore, these results supply new evidence in the study of the relationship between environmental adaptation and virulence mechanisms by the regulation of sRNA in *V. alginolyticus.* However, more work needs to be done to find the precise target genes of *srvg23535* and to fully understand the regulatory mechanism, thereby providing new antibacterial therapeutic targets or attenuated vaccine targets against *Vibrio* spp.

## Conclusion

This study comprehensively identified and characterized a novel sRNA *srvg23535* in *V. alginolyticus*. It is conserved among several *Vibrio* spp. with four stem-loop structures. Furthermore, according to the PM analysis, *srvg23535* affects the utilization of several carbon and nitrogen sources and even the response to some stress conditions. Although target prediction was conducted, not all the predicted targets are consistent with the phenotypes. As a matter of future study, we recommend that the interaction of *srvg23535* and the predicted target genes are verified, and their expression is compared by RNA-seq to assess the genotypic regulation.

## Author Contributions

YD conceived the study, analyzed the data, and wrote the manuscript. CHC, HM, and JW performed the experiments. YS, SL, and ZG critically revised the manuscript. JF and CC contributed the reagents. All authors read and approved the manuscript for publication.

## Conflict of Interest Statement

The authors declare that the research was conducted in the absence of any commercial or financial relationships that could be construed as a potential conflict of interest.

## Supplementary Material

FIGURE S1 The original gels of RACE results. **(A)** PCR results of 5′-RACE. Lane M: DNA Marker DL2000; Lane 3: 5′-RACE of *srvg23535*; Remaining lanes are 5′-RACE results of other sRNAs those have been done at the same time. **(B)** PCR results of 3′-RACE. Lane M: DNA Marker DL2000; Lane 6: 3′-RACE of *srvg23535*; Remaining lanes are 3′-RACE results of other sRNAs those have been done at the same time.

FIGURE S2 **(A–H)** Effect of *srvg23535* deletion on the utilization of 95 various carbon sources which were test by the PM01 plate (the results are represented as the average values, *n* = 3).

FIGURE S3 **(A–H)** Effect of *srvg23535* deletion on the utilization of 95 various nitrogen sources which were test by the PM03 plate (the results are represented as the average values, *n* = 3).

FIGURE S4 **(A–H)** Effect of *srvg23535* deletion on the growth responses to osmolytes stress which were test by the PM09 plate (the results are represented as the average values, *n* = 3).

FIGURE S5 **(A–H)** Effect of *srvg23535* deletion on the growth responses to different pH stress which were test by the PM10 plate (the results are represented as the average values, *n* = 3).

TABLE S1 Nucleotide sequences of primers or probes used in this study.

TABLE S2 Tested components of the PM01, PM03, PM09, and PM10 plates.

TABLE S3 Details of common targets predicted by IntaRNA and target RNA2.

TABLE S4 Details of phenotype differences generated by PM technology (the results of the graphs are represented as the average ± SE, *n* = 3).

FILE S1 PM Procedures for *E. coli* and other GN Bacteria.

FILE S2 The original data of phenotype differences.
