# Corrigendum: Identification of a Novel Small RNA *srvg23535* in *Vibrio alginolyticus* ZJ-T and Its Characterization With Phenotype MicroArray Technology

**DOI:** 10.3389/fmicb.2019.00021

**Published:** 2019-01-31

**Authors:** Yiqin Deng, Youlu Su, Songlin Liu, Zhixun Guo, Changhong Cheng, Hongling Ma, Jinjun Wu, Juan Feng, Chang Chen

**Affiliations:** ^1^Key Laboratory of South China Sea Fishery Resources Exploitation and Utilization, Ministry of Agriculture and Rural Affairs, South China Sea Fisheries Research Institute, Chinese Academy of Fishery Sciences, Guangzhou, China; ^2^Key Laboratory of Tropical Marine Bio-resources and Ecology, Guangdong Provincial Key Laboratory of Applied Marine Biology, South China Sea Institute of Oceanology, Chinese Academy of Sciences, Guangzhou, China; ^3^Key Laboratory of Tropical Marine Bio-resources and Ecology, South China Sea Institute of Oceanology, Chinese Academy of Sciences, Guangzhou, China; ^4^Xisha/Nansha Ocean Observation and Research Station, South China Sea Institute of Oceanology, Chinese Academy of Sciences, Guangzhou, China

**Keywords:** small non-coding RNAs, *srvg23535*, *Vibrio alginolyticus*, identification, Phenotype MicroArray technology

In the published article, there was an error in affiliation 1. Instead of “Ministry of Agriculture” the correct name of the ministry is “Ministry of Agriculture and Rural Affairs”.

In Table 1, the references for “53813,” “GEB88,” and “pSW7848,” were incorrectly written as “This lab.” It should be “Le Roux et al., 2007,” “Nguyen et al., 2018,” and “Val et al., 2012,” respectively. Additionally, the intermediate host *Escherichia coli* strain was named as “GEB802,” but should be “53813.”

The corrected Table 1 appears below.

**Table 1:** Strains and plasmids used in this study.

**Strains or plasmids**	**Relevant characteristics**	**Sources**
***V. alginolyticus***		
ZJ-T	Ap^r^ (ampicillin resistant), translucent/smooth variant of wild strain ZJ-51 (Xiaochun et al., 2017); isolated from diseased *Epinephelus coioides* off the Southern China coast	Chang et al., 2009
ZJ-T-Δ*srvg23535*	Apr; ZJ-T carrying an deletion of *srvg23535*	This study
***E. coli***		
Π3813	Emr^r^, Tc^r^, *lacIQ*, *thi1*, *supE44*, *endA1*, *recA1*, *hsdR17*, *gyrA462*, *zei298::tn10[Tc]*, Δ*thyA*:: *(erm-pir116)*; the intermediate host of suicide vector pSW7848	Le Roux et al., 2007
GEB883	Ery^r^, Tet^r^, WT *E. coli* K12 Δ*dapA*::*erm pir RP4-2* Δ*recA gyrA462*, *zei298*::*Tn10*; donor strain for conjugation	Nguyen et al., 2018
***Plasmids***		
pSW7848	Cmr; suicide vector with an R6K origin, requiring the Pir protein for its replication, and the *ccdB* toxin gene	Val et al., 2012
pSW7848-Δ*srvg23535*	Cmr; pSW848 containing the mutant allele of Δ*srvg23535*	This study

A correction has also been made to the **MATERIALS AND METHODS, Bacterial Strains, Plasmids, and Growth Conditions and Gene Disruption**, paragraph one:

“To generate the sRNA disruptant, the sequence from 46 bp before the 5′ end to 2 bp after the 3′ end was deleted from the chromosome of *V. alginolyticus* ZJ-T. The deletion was constructed by homologous recombination as described before with some modification (Yiqin et al., 2016). Briefly, two flanking fragments of *srvg23535* (Figure 1A) were amplified with two pairs of primers, *srvg23535*-UP-F and -R and *srvg23535*-DOWN-F and -R respectively, and the linearized pSW7848 was amplified with pSW7848-F and -R (Supplementary Table 1). *srvg23535*-UP-F and *srvg23535*-DOWN-R contained overlapping extensions with pSW7848-R and -F, respectively, and *srvg23535*-UP-R contained overlapping extensions with *srvg23535*-DOWN-F. The two flanking fragments were further assembled into the linearized pSW7848 by using a ClonExpress Multis One Step Cloning Kit (Vozyme, China), generating the recombinant plasmid pSW7848-Δ*srvg23535* comprising the 1,084 bp upstream and 1,105 bp downstream regions of *srvg23535* (Table 1), using *E. coli* Π3813 as an intermediate host. The recombinant plasmid was transferred by conjugation from strain GEB883 (Table 1) to *V. alginolyticus* ZJ-T before allelic exchange as described above. The sRNA disruptant was then confirmed by sequencing and the strain was named ZJ-T-Δ*srvg23535* (Figure 1 and Table 1).”

The authors apologize for these errors and state that this does not change the scientific conclusions of the article in any way. The original article has been updated.

## Conflict of Interest Statement

The authors declare that the research was conducted in the absence of any commercial or financial relationships that could be construed as a potential conflict of interest.
