# Supplementary figures and images for: Identification of a Novel Small RNA srvg23535 in Vibrio alginolyticus ZJ-T and Its Characterization With Phenotype MicroArray Technology
Source: Front Microbiol. 2018 Oct 5;9:2394. doi: 10.3389/fmicb.2018.02394 (PMC6186989; doi:10.3389/fmicb.2018.02394)

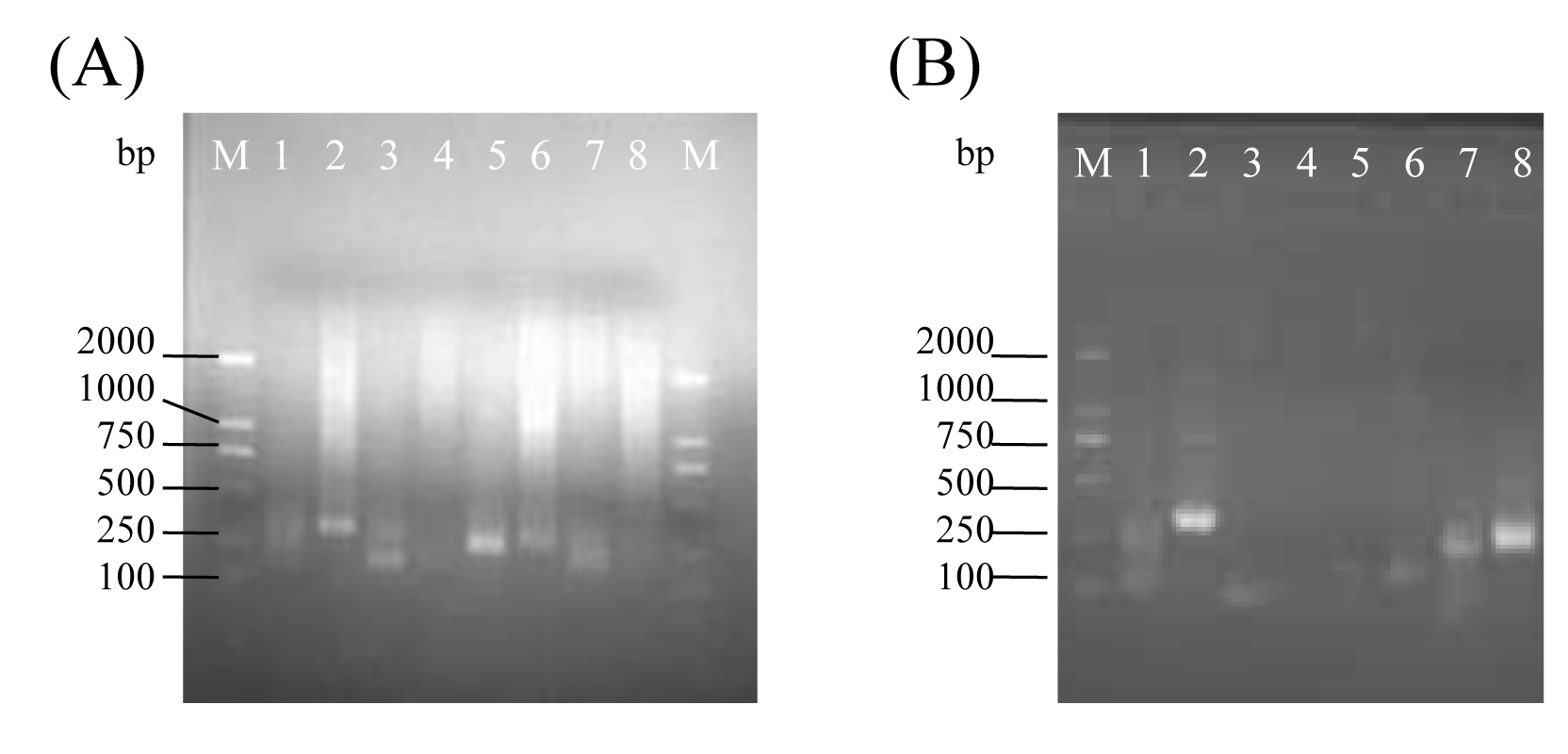

Supplement: FIGURE S1 — The original gels of RACE results. (A) PCR results of 5′-RACE. Lane M: DNA Marker DL2000; Lane 3: 5′-RACE of srvg23535; Remaining lanes are 5′-RACE results of other sRNAs those have been done at the same time. (B) PCR results of 3′-RACE. Lane M: DNA Marker DL2000; Lane 6: 3′-RACE of srvg23535; Remaining lanes are 3′-RACE results of other sRNAs those have been done at the same time. [file Image_1.TIF]

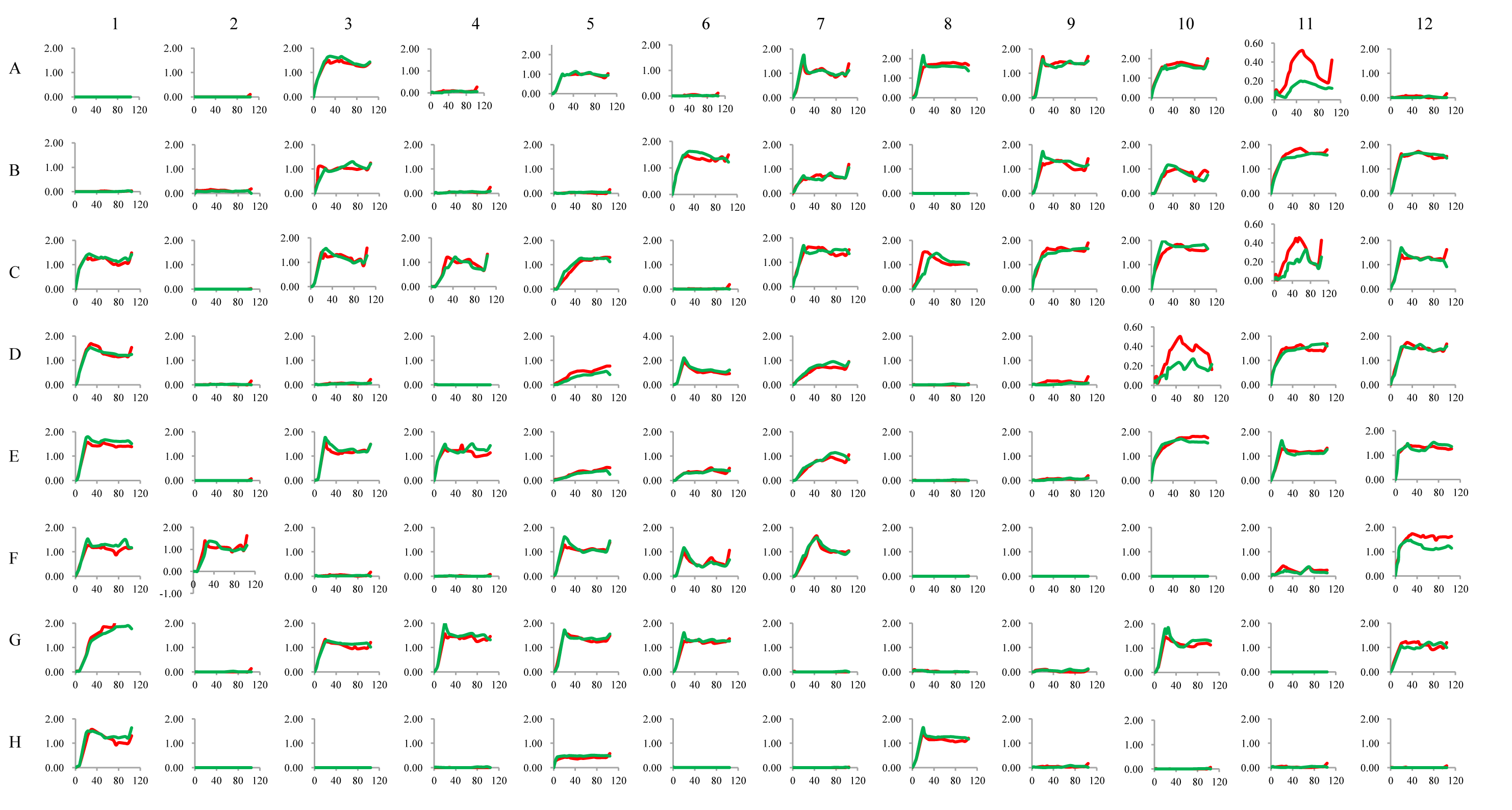

Supplement: FIGURE S2 — (A–H) Effect of srvg23535 deletion on the utilization of 95 various carbon sources which were test by the PM01 plate (the results are represented as the average values, n = 3). [file Image_2.TIF]

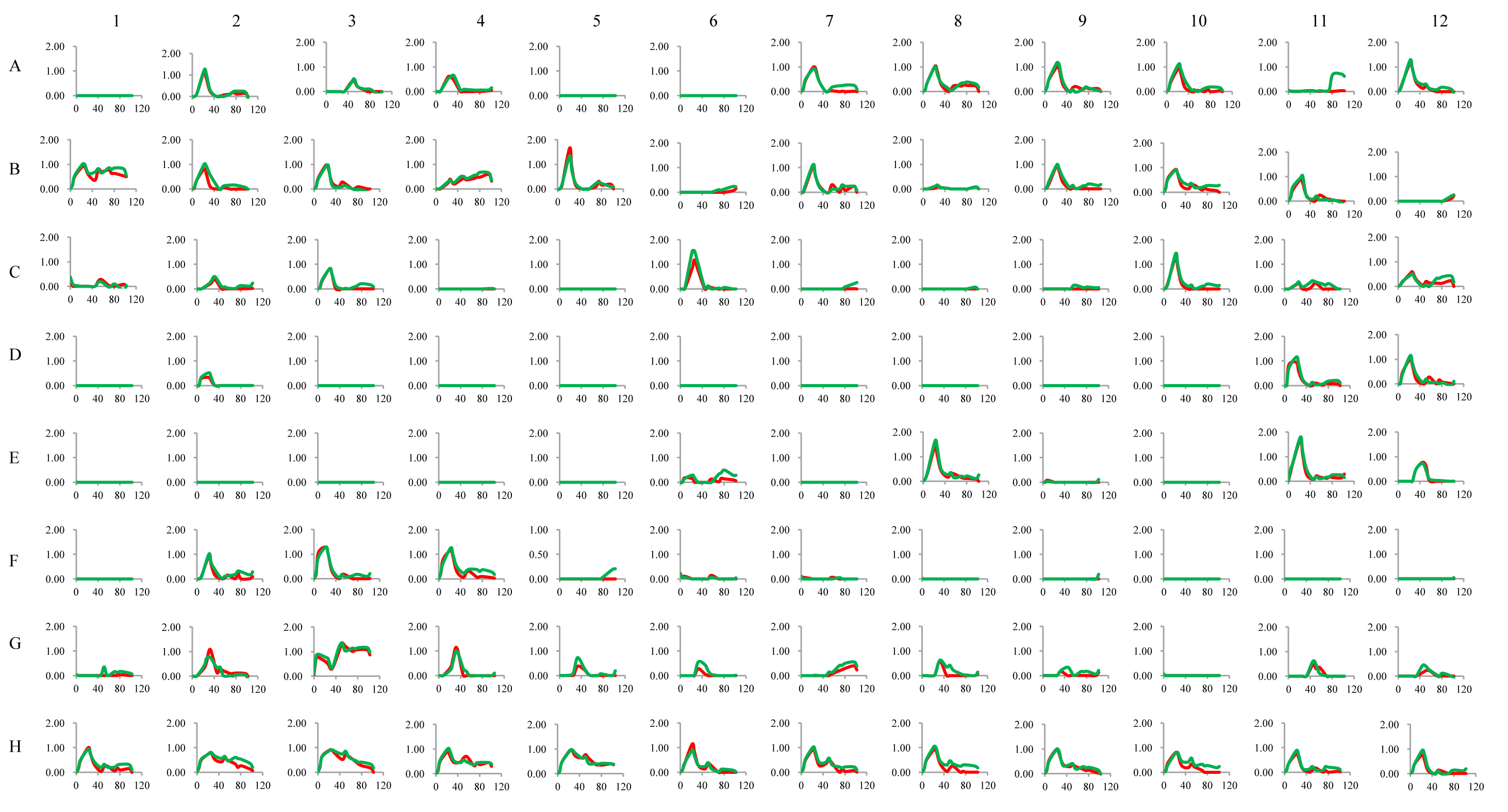

Supplement: FIGURE S3 — (A–H) Effect of srvg23535 deletion on the utilization of 95 various nitrogen sources which were test by the PM03 plate (the results are represented as the average values, n = 3). [file Image_3.TIF]

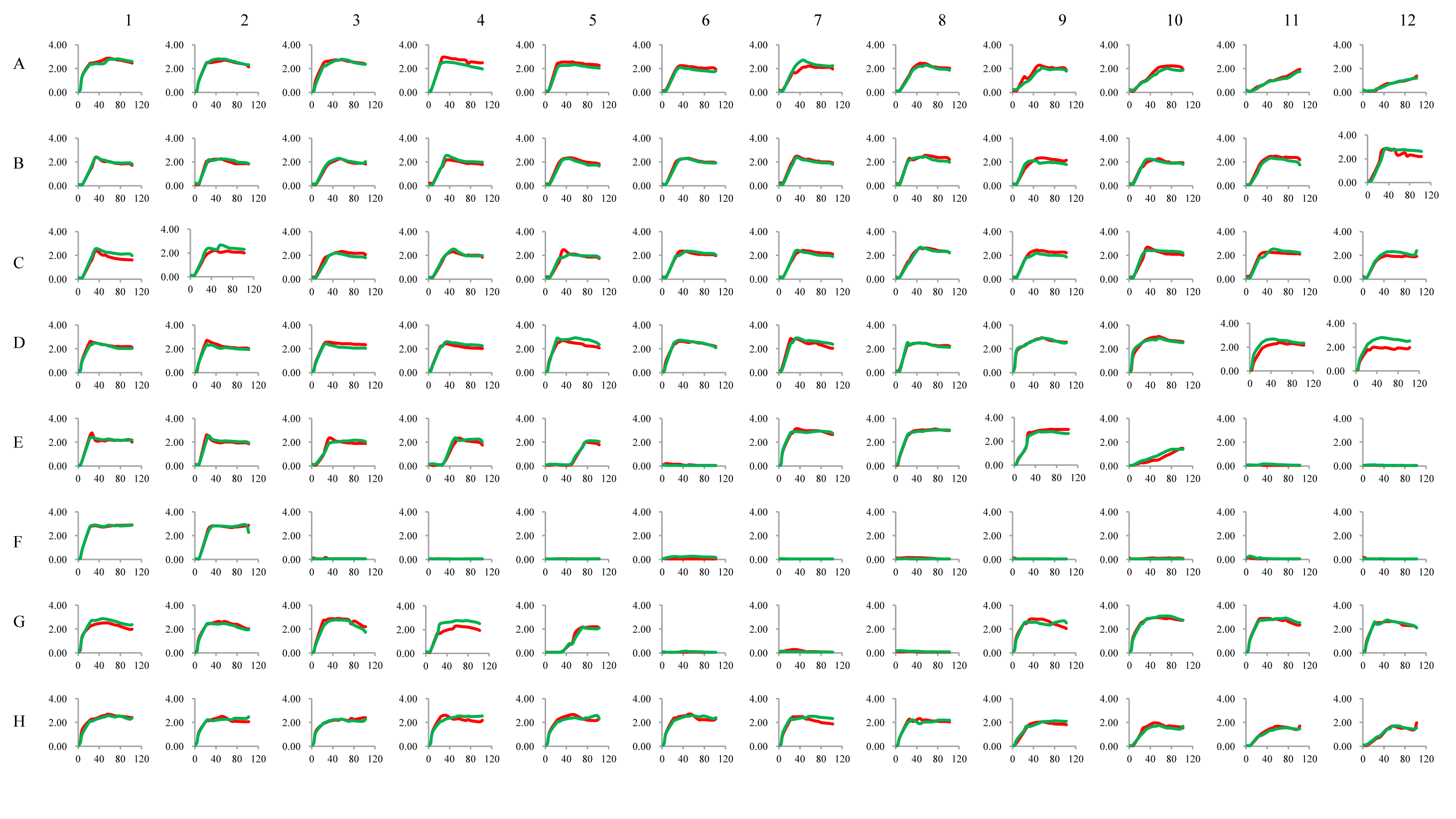

Supplement: FIGURE S4 — (A–H) Effect of srvg23535 deletion on the growth responses to osmolytes stress which were test by the PM09 plate (the results are represented as the average values, n = 3). [file Image_4.TIF]

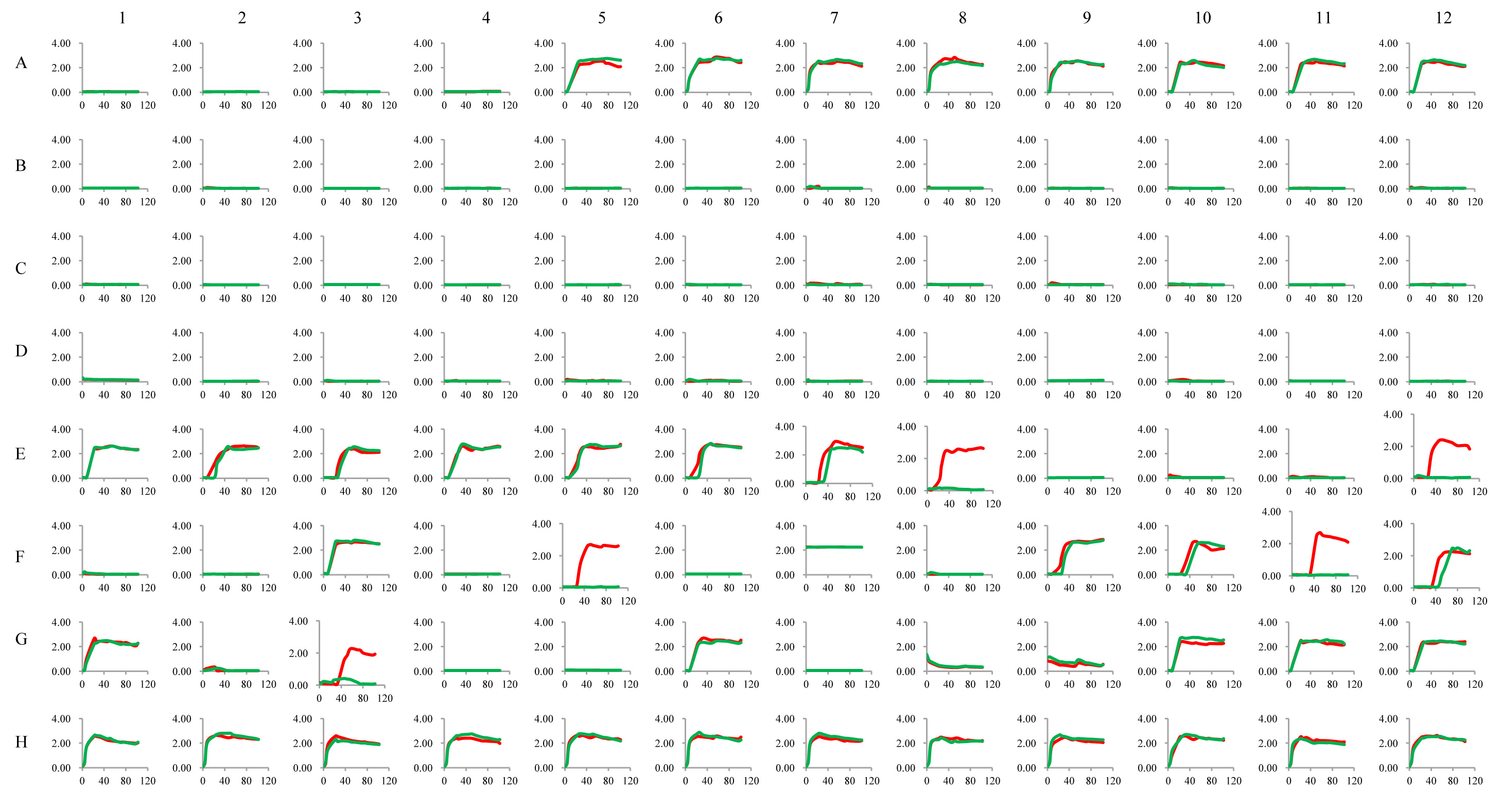

Supplement: FIGURE S5 — (A–H) Effect of srvg23535 deletion on the growth responses to different pH stress which were test by the PM10 plate (the results are represented as the average values, n = 3). [file Image_5.TIF]
