# Supplementary material for: Identification of a Novel Small RNA srvg23535 in Vibrio alginolyticus ZJ-T and Its Characterization With Phenotype MicroArray Technology
Source: Front Microbiol. 2018 Oct 5;9:2394. doi: 10.3389/fmicb.2018.02394 (PMC6186989; doi:10.3389/fmicb.2018.02394)
Supplement: FILE S2 — The original data of phenotype differences. [file Data_Sheet_1.PDF]

PM Procedures for *E. coli* and other GN Bacteria

SECTION I: MATERIALS

Section A. List of Equipment, Chemicals and Materials

Table 1: Equipment

Equipment	Source	Catalog #
OmniLog PM	Biolog	91171
Turbidimeter	Biolog	3531
Multichannel Pipetter	Biolog	3501A

Table 2: Chemicals and Materials for Inoculation Procedure

Chemicals and Materials	Source	Catalog #
PM panels (PM 1 – 20)	Biolog	12111,12112,12121,<br>12131,12141,12181,<br>12182,12183,12161,<br>12162,12211-12220
IF-0 GN Base inoculating fluid (1.2x)	Biolog	72256
IF-10 GN Base inoculating fluid (1.2x)	Biolog	72254
Dye mix A (100x)	Biolog	74221
BUG+B agar plates	Biolog	71102
sterile cotton swabs	Biolog	3021
sterile pipet tips	Biolog	3001
sterile reservoirs	Biolog	3002
sterile 20 x 150 test tubes	E+K Scientific	266B
sterile 120 ml plastic vial	Capitol Vial Corp.	1-24-786
sealing tape for microplates (optional)	Phenix Research Products	LMT-SEAL-EX
sodium succinate	Sigma	S2378
ferric citrate	Sigma	F6129
sodium pyruvate	Sigma	P2256

Section B. Additions to Inoculating Fluids

Table 3: Stock Solutions Added to PM Inoculating Fluids

**Carbon Source Stock Solution**	**Concen-tration**	**Formula Weight**	**Grams/ 100ml**	**Concen-tration Factor**	**Sterilized by**	**Storage Temp**	**Notes**
sodium succinate/<br>ferric citrate	2 M<br>200 µM	270.1<br>244.9	54.02<br>4.9(mg)	100x	Filtration	4°C	
sodium pyruvate	2 M	110	22.0	100x	Filtration	4°C	

Table 4: Ingredients and Final Concentrations in PM Inoculating Fluids with the Cell Suspensions

**Ingredient**	**PM 1 – 2**	**PM 3 – 8**	**PM 9 – 20**
IF-0 GN	+	+	-
IF-10 GN	-	-	+
Dye Mix A	+	+	+
sodium succinate/ferric citrate	-	20mM/2uM	-
cell density	85% T	85% T	1:200 of 85% T

Alternative protocol using pyruvate instead of succinate

**Ingredient**	**PM 1 – 2**	**PM 3 – 8**	**PM 9 – 20**
IF-0 GN	+	+	-
IF-10 GN	-	-	+
Dye Mix A	+	+	+
sodium pyruvate	-	20mM	-
cell density	85% T	85% T	1:200 of 85% T

Additional Comments:

1. The standard protocol uses sodium succinate as the carbon source in PM 3-8. Other carbon sources such as sodium pyruvate or glucose can be substituted for succinate. The N, P, and S utilization patterns will probably change with different carbon sources due to catabolite repression effects. *Yersinia* will not use succinate as the carbon source, so pyruvate is recommended as the best alternative.
2. These arrays have NH_4^+, PO_4^{-3}, and SO_4^{-2} as the N-, P-, and S-sources. If the strain requires an alternative N-, P-, or S-source or supplemental concentration, the supplement needs to be added to the appropriate inoculating fluid(s).
3. *Yersinia, Proteus,* and *Obesumbacterium* strains usually require a reduced form of sulfur added to all inoculating fluids. We recommend adding 1mM sodium thiosulfate.
4. If the strain has auxotrophic requirements, these need to be defined and added to the inoculating fluids for PM 1-8. For most nutrients such as amino acids, purines, pyrimidines, etc., 50 µM is usually sufficient. For most vitamins this can be lowered to 0.5 µM.

SECTION II: PROCEDURES for PM 1 – 20 Inoculation

Section A. Cell Suspension Preparation and PM Inoculation

Preparation of PM Inoculating Fluids

1. Prepare IF-0 by adding 25 ml of sterile water into the bottle containing 125 ml of 1.2x IF-0.
2. Pipet 16 ml of this IF-0 into a 20 x 150 mm sterile capped test tube.
3. Prepare IF-0+dye by adding 1.8 ml of dye mix and 23.2 ml of sterile water into the bottle containing 125 ml of 1.2x IF-0.
4. Pipet 75 ml of this IF-0+dye into a 120 ml sterile plastic vial.
5. Prepare IF-10+dye by adding 1.5 ml of dye mix and 23.5 ml of sterile water into the bottle containing 125 ml of 1.2x IF-10.
6. Pipet 120 ml of this IF-10+dye into a 120 ml sterile plastic vial.

Inoculation of PM Panels (see procedure diagramed in Fig. 1)

Step 1 - 2: Prepare Cell Suspensions

1. Grow the strain on a BUG+B agar plate by streaking the bacterium for isolated colonies and allowing it to grow overnight at 30°-37°C. Subculture a second time if the cells were streaked from a frozen culture stock. Remove cells from the BUG+B agar plate using a sterile swab and transfer into the sterile capped tube containing 16 ml IF-0. Stir the cell suspension with the swab to obtain a uniform suspension. Check the turbidity of the suspension and add cells to achieve 42% T (transmittance) in the Biolog Turbidimeter.
2. Add 15 ml of 42%T cell suspension to the vial containing 75 ml of IF-0+dye (1:5 dilution). Mix completely but gently. DO NOT create air bubbles in the cell suspension. The final cell density is 85% T.

Step 3 - 5: Inoculate PM 9 – 20

3. Transfer 600 µl of the 85% T cell suspension prepared in step 2 into the sterile, pre-filled vial containing 120 ml IF-10+dye (1:200 dilution). Mix completely but gently. DO NOT create air bubbles in the cell suspension.
4. Transfer this cell suspension into a sterile reservoir.
5. Inoculate PM 9 – 20 with this cell suspension, 100 µl / well.

Step 6 - 7: Inoculate PM 1 – 2

6. Transfer 22 ml of the 85% T cell suspension prepared in step 2 into a sterile reservoir.
7. Inoculate PM 1 – 2 with this cell suspension, 100 µl / well.

Step 8 - 10: Inoculate PM 3 – 8

8. Add 680 µl of the 2M sodium succinate/200uM ferric citrate (100X) to the remaining 68 ml of the 85% T cell suspension prepared in step 2.

9. Transfer this cell suspension into a sterile reservoir.
10. Inoculate PM 3 – 8 with this cell suspension, 100 μl / well.

Figure 1. PM Procedures for *E. coli* and other GN Bacteria

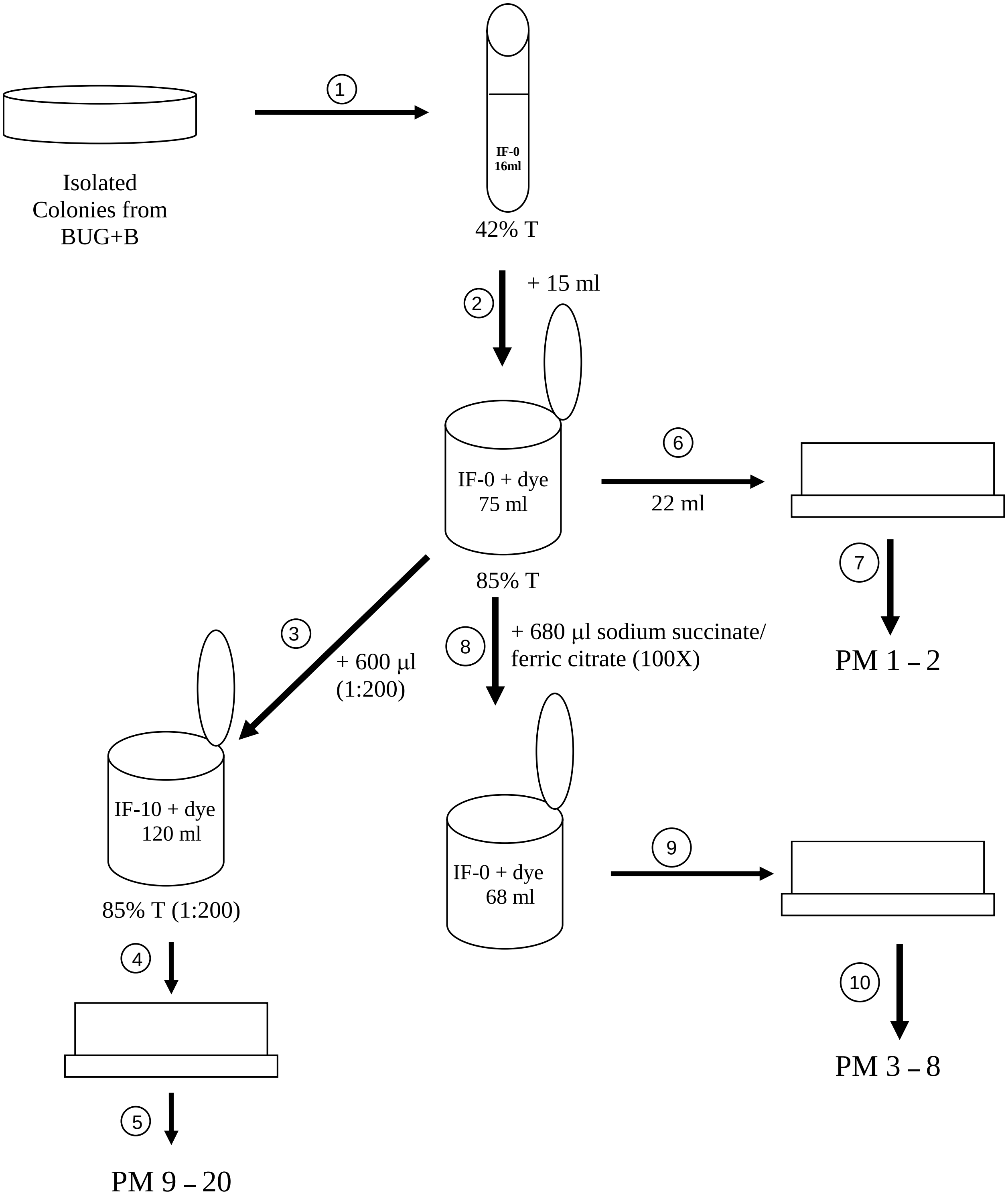

Section B. Incubation and Data Collection

1. Enter worksheet data into OmniLog Software.
2. Load the OmniLog.
3. Incubate all PMs in the OmniLog at **30°-37°C**. Incubate all PMs for **24 to 48** hours
4. Remove plates from OmniLog and store at 4°C.
5. Collect the data for analysis.
